# Gene Expression Profiling Predicts Sensitivity of Chronic Lymphocytic Leukemia Cells to Dasatinib

**DOI:** 10.1097/HS9.0000000000000514

**Published:** 2020-12-16

**Authors:** Tamara J. Blätte, Marcin M. Machnicki, Eliza Glodkowska-Mrowka, Anna Dolnik, Marta Karp, Agnieszka Karczmarczyk, Krzysztof Giannopoulos, Lars Bullinger, Tomasz Stoklosa

**Affiliations:** 1 Department of Internal Medicine III, University Hospital of Ulm, Germany; 2 Department of Hematology, Oncology and Tumor Immunology, Charité University Medicine, Berlin, Germany; 3 Department of Tumor Biology and Genetics, Medical University of Warsaw, Poland; 4 Department of Experimental Hematology, Institute of Hematology and Transfusion Medicine, Warsaw, Poland; 5 Department of Experimental Hematooncology, Medical University of Lublin, Poland.

While targeted therapy in chronic lymphocytic leukemia (CLL) has significantly improved prognosis for many patients, overall outcome remains heterogeneous. Additional therapeutic options are particularly needed for (1) patients experiencing drug toxicities or resistance and (2) patients residing in countries whose healthcare system has not yet approved or does not cover the costs of these new treatments.^1,2^

The present study, therefore, focused on dasatinib, a second-generation tyrosine kinase inhibitor (TKI) of breakpoint cluster region-Abelson1, Sarcoma-family kinases, and also Bruton’s tyrosine kinase, that was approved for the treatment of chronic myeloid leukemia in 2006 and for which recently generics have become available.^3^ Dasatinib represents a promising candidate for potential next-line therapy in CLL, as CLL cells tend to be both dependent on B-cell receptor signaling for survival and highly responsive to Bruton’s tyrosine kinase targeting via ibrutinib.^4^ Moderate results from previous clinical trials in relapsed/refractory CLL suggest that predictive biomarkers are needed to identify those patients who may benefit most from dasatinib.^5-9^ In this study, we therefore used RNA sequencing to characterize the gene-expression profiles of CLL cells sensitive and resistant to dasatinib and define a specific gene-expression signature associated with drug sensitivity. Sample and method details are provided in the supplementary information (http://links.lww.com/HS/A124), raw and normalized gene expression data are accessible through Gene Expression Omnibus (accession number GSE151159).

To determine the spectrum of in vitro sensitivity of primary CLL cells to dasatinib, we isolated peripheral blood lymphocytes (PBLs) from newly diagnosed, previously untreated patients (n = 16, Table S1, http://links.lww.com/HS/A124) and performed cytotoxicity assays. In short, PBLs were exposed for 48 hours to 180 nM dasatinib, reflecting plasma drug concentrations observed in a clinical setting,^10^ and subsequent XTT and CellTiter-Blue assays were used to assess cell viability. Based on the cytotoxicity assay results (Figure 1A and Figure S1, http://links.lww.com/HS/A124), we split patients into two groups, responders (n = 7) and nonresponders (n = 9), using an arbitrarily chosen cutoff of 50% viability after dasatinib treatment. The average viability in responders and nonresponders was 26% (range 11%-37%) and 75% (range 51%-94%), respectively (unpaired one-tailed *t* test; *P* < 0.0001). Notably, patients who classified as responders presented with clinical features of poor prognosis more frequently than nonresponders. Specifically, responders were associated with a more advanced stage of the disease, higher lymphocyte counts, and a higher frequency of unmutated immunoglobulin heavy chain variable status (Table S1, http://links.lww.com/HS/A124).

**Figure 1.**
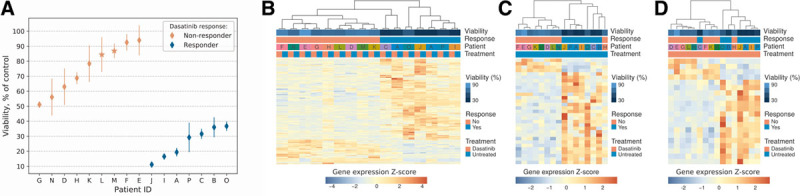
**Gene expression signatures in responders and nonresponders to dasatinib.** (A), Viability of CLL cells isolated from responders (n = 7) and nonresponders (n = 9), treated with 180 nM dasatinib. Error bars represent a fraction of standard deviation in treated cells vs mean viability measurement in corresponding controls. XTT assay results are shown in the chart except for patients L and M (marked with asterisks) for whom XTT assay was inconclusive, and CellTiter-Blue assay results are shown. B–D, Hierarchical clustering of RNA sequencing data of all dasatinib-treated and dasatinib-untreated samples (B), all untreated samples only (C) and all treated samples only (D), each based on the Pearson correlation of DEGs between responders (n = 7) and nonresponders (n = 9). Heatmaps show row-/gene-wise *Z* scores; columns correspond to individual samples. Viability refers to the average cell viability observed in the in vitro cytotoxicity assays. Responders and nonresponders share distinct gene-expression signatures, both before and after treatment. CLL = chronic lymphocytic leukemia; DEG = differentially expressed gene; ID = patient identifier.

To determine whether responders and nonresponders were associated with distinct gene expression signatures, we performed RNA sequencing of all patients’ treated and untreated samples on the Illumina HiSeq2000. We then used DESeq2 to test for differentially expressed genes (DEGs) between responders and nonresponders, with an absolute fold change of at least 1.5 and a false discovery rate of at most 0.1. We detected 154 such DEGs (Figure 1B and Table S2, http://links.lww.com/HS/A124). This initial comparison included all samples, both treated and untreated. However, since patients had been labeled as responders/nonresponders based on the treated cells’ phenotype, treated responders and nonresponders were naturally expected to differ substantially. Of real interest was rather the question whether responders differed from nonresponders per se, even before the drug was added. We therefore performed the same analysis for the treated and untreated samples separately. There, we found 31/154 DEGs between the untreated responders and nonresponders (Figure 1C and Table S2, http://links.lww.com/HS/A124) and 20/154 DEGs in the analogous comparison of the treated samples (Figure 1D and Table S2, http://links.lww.com/HS/A124). Sixteen DEGs were shared between all three comparisons (Table S2, http://links.lww.com/HS/A124).

To compare gene expression differences at the pathway level, we performed gene set enrichment analyses using gene set enrichment analysis and molecular signatures database hallmark gene sets.^11^ We found that both treated and untreated responders were associated with increased stress signaling (apoptosis, p53, unfolded protein/reactive oxygen species/ultraviolet responses up), metabolic signaling (glycolysis, mammalian target of rapamycin complex 1 up), and immunologic signaling (inflammatory, complement, interleukins, interferons, tumor necrosis factor signaling up). In addition, treated but not untreated responders displayed an increased expression of genes implicated in oxidative phosphorylation and peroxisome signaling, allograft rejection, xenobiotic metabolism, and DNA repair. We thus found a gene expression signature distinguishing responders and nonresponders both before and after treatment. In general, responder PBLs seemed to be more stressed and more active than PBLs of nonresponders, both metabolically and immunologically, and treatment appeared to further augment these differences (Figure 2A).

**Figure 2.**
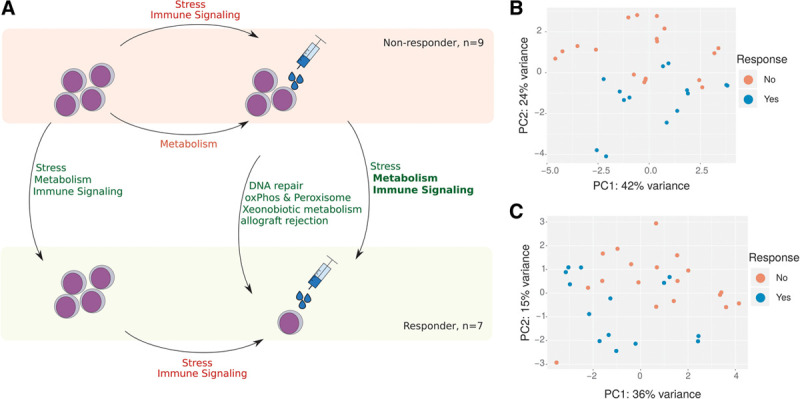
**Key pathways associated with response to dasatinib.** (A), Schematic overview of the pathway level gene expression differences between responders and nonresponders, and treated and untreated cells. Results are based on GSEA analyses of the MSigDB Hallmark collection: Green pathways indicate related gene sets that tended to be upregulated in the respective group (arrowhead) relative to one of the others (arrow tail). Analogously, red pathways indicate downregulated gene sets. B and C, PCs 1 and 2 from PCA of all dasatinib-treated and dasatinib-untreated samples for genes of the MSigDB hallmark collection gene sets HALLMARK_NOTCH_SIGNALING (B) and HALLMARK_DNA_REPAIR (C). Responders and nonresponders share distinct gene-expression signatures involving both pathways. GSEA = gene set enrichment analysis; MSigDB = molecular signatures database; PC = principal component; PCAs = principal component analyses.

To study the effect of treatment in more detail, we accounted for inter-individual heterogeneity in DESeq2’s regression model by performing a paired analysis to separately compare the treated versus untreated sample of responders and nonresponders. We detected 101 and 92 DEGs, respectively, and found that treated samples of both responders and nonresponders were associated with decreased stress signaling (apoptosis, p53, unfolded protein response down) and decreased immunologic signaling (allograft rejection, coagulation, complement, inflammatory response, interleukins, interferons, tumor necrosis factor signaling down). In addition, in treated but not untreated nonresponders, we found decreased metabolic signaling, as indicated by the decreased expression of genes implicated in adipogenesis, fatty acid metabolism, oxidative phosphorylation, and glycolysis (Figure 2A).

We then checked whether differences between responders and nonresponders might be explained by differences at the genetic level. Specifically, we checked our samples for copy number variations and gene mutations in *ATM*, *BIRC3*, *MYD88*, *NOTCH1*, *SF3B1*, and *TP53*, which are all generally recurrent in CLL.^12^ With the exception of *MYD88* and *TP53* mutations, each of these aberrations was found in at least one sample, though none were notably recurrent (Tables S3 and S4, http://links.lww.com/HS/A124). We hypothesized that the associated signaling pathways might nonetheless be differentially deregulated at the RNA level. Accordingly, we performed two principal component analyses (PCAs) of all our samples, based on those genes that make up the molecular signatures database HALLMARK_NOTCH_SIGNALING and HALLMARK_DNA_REPAIR gene sets, respectively (Figure 2, B and C). While a perfect linear segregation was not possible in either case, samples clearly did separate into responders and nonresponders on the first 2 principal components, indicating that the differential expression of these signaling pathways was not limited to the individual mutated cases and may instead represent a defining feature of our groups in general.

The main limitation of our study’s potential predictive value is its small sample size. We therefore validated our identified gene expression signature using the Library of Integrated Network-Based Cellular Signatures L1000 database,^13^ which contains thousands of independent, drug-induced gene expression profiles (GEPs) from various cell lines, compounds, doses, and time points. Indeed, when queried for GEPs correlated with the “dasatinib treatment” signature, which we had identified in the comparison of treated versus untreated samples, the database returned several TKIs, and dasatinib itself was even among the top hits (Figure S2A, http://links.lww.com/HS/A124). We then repeated this experiment for our “dasatinib response” signature from the responder versus nonresponder comparison and identified multiple compounds predicted to increase treatment sensitivity to dasatinib that may warrant follow-up investigations (Figures S2B and S3, http://links.lww.com/HS/A124).

Thus, our study serves as an important proof-of-principle, providing additional evidence that (1) dasatinib may represent a viable therapeutic option in a group of CLL patients and (2) GEP-based markers can be used to predict response to TKI therapy in CLL and identify these patients before any treatment is given at all. Notably, such predictive signatures might also be generated for other kinase inhibitors currently approved for CLL treatment, similar to the BCL-2 homology domain 3 profiling recently suggested as a biomarker of patient response to venetoclax.^14^ This is in line with a recent study on ibrutinib that also suggests the feasibility of transcriptome-based treatment monitoring in CLL.^15^ In the future, respective markers could be used to indicate not only resistance to novel treatment strategies but also response to alternative drugs such as dasatinib, perhaps in combination with other “repurposed” drugs capable of reverting respective leukemia signatures. While our study was based on only a limited number of treatment-naïve CLL patient samples, follow-up studies of larger, as well as more selected cohorts such as relapsed/refractory patients with failed first-line (ibrutinib) therapy are warranted. Novel gene expression profiling technologies applied in clinical routine, such as the Nanostring technology, could ensure translation of respective biomarkers into the clinic.

## Sources of funding

This work was supported by a grant from the Polish National Science Center N N402 676540 (TS) and in part by European Union program: FP7-REGPOT-2012-CT2012-316254-BASTION (twinning visits).

## Disclosures

LB involved in the advisory role or expert testimony from Bristol-Myers Squibb, Celgene, Daiichi Sankyo, Gilead, Hexal, Janssen, Jazz Pharmaceuticals, Menarini, Novartis, and Pfizer; received Honoraria from Abbvie, Amgen, Astellas, Bristol-Myers Squibb, Celgene, Daiichi Sankyo, Janssen, Jazz Pharmaceuticals, Novartis, Pfizer, Sanofi, and Seattle Genetics; and received financing of scientific research from Bayer. TS received Honoraria from Janssen. The remaining authors have indicated they have no potential conflicts of interest to disclose.

## Supplementary Material


