# Supplementary material for: Gene Expression Profiling Predicts Sensitivity of Chronic Lymphocytic Leukemia Cells to Dasatinib
Source: Hemasphere. 2020 Dec 16;5(1):e514. doi: 10.1097/HS9.0000000000000514 (PMC7746204; doi:10.1097/HS9.0000000000000514)

**Supplementary Materials & Methods**

**Patient samples**

We used CLL patients’ blood samples obtained after informed consent. All patients were diagnosed at the Medical University of Lublin with CLL and were naive to antileukemic treatment. Patients were clinically and molecularly characterized, including staging according to Rai classification, IGVH mutation status and other prognostic factors (clinical characteristics of patients are presented in Supplementary Table S1). The study was approved by the local ethics committee of the Medical University of Lublin with the number KE-0254/254/2012.

**Cell isolation**

Peripheral blood lymphocytes (PBL) were isolated from fresh blood by density gradient separation. Blood was diluted with PBS at a 1:1 ratio, layered over Histopaque 1077 (Sigma-Aldrich), and centrifuged at 700rcf for 30min at room temperature. The top layer containing mononuclear cells was gently aspirated, washed with PBS with two centrifugation steps at 700rcf and 400rcf for 10min and cultured in IMDM medium (Gibco) without phenol red, supplemented with 10% HyClone SH30072.03 calf serum (FBS, GE Healthcare) and 1x antibiotic/antimycotic-solution 30-004-Cl (Corning). Cells were cryopreserved in liquid nitrogen in culture media supplemented with 50% FBS and 10% DMSO (D8418 Sigma-Aldrich).

**Immunophenotyping (CD5 expression)**

The immunophenotypic analysis was performed by flow cytometry. The standard diagnostic flow cytometric analysis included monoclonal antibodies (MoAbs) anti-CD5-FITC/CD19-PE (BD Biosciences, USA). A standard, whole-blood assay with erythrocyte cell lysis was used for preparing the peripheral blood specimens. The samples were analyzed by flow cytometry directly after preparation. For data acquisition and analysis, a FACSCalibur instrument (Becton Dickinson, USA) with CellQuest software (Becton Dickinson, USA) was used. For each analysis 10,000 events were acquired and analyzed. Non-viable cells and debris were excluded based on forward and side scatter. The percentage of positive cells was measured from a cut-off set using isotype-matched nonspecific control antibody.

**Dasatinib sensitivity assay**

For viability testing, freshly isolated or previously cryopreserved cells were seeded into flat-bottom, untreated 96-well plates in tetraplicates or at least triplicates, if enough cells were not available. Cells were cultured for 2h in 100µl of IMDM medium prior to dasatinib treatment. Subsequently, control and treatment groups were created by adding either IMDM or dasatinib diluted with IMDM to a final concentration of 180nM, relevant to clinical pharmacokinetics of dasatinib.^1^

Dasatinib sensitivity was measured after 48h treatment using XTT (2,3-Bis-(2-Methoxy-4-Nitro-5-Sulfophenyl)-2H-Tetrazolium-5-Carboxanilide) (Invitrogen) and CellTiter-Blue assays with cell concentrations set at 0.5 and 0.3 × 10^6^ cells per well, respectively. XTT was added to a final concentration of 0.29mg/ml, supplemented with phenazine methosulfate (P9625, Sigma-Aldrich) at a final concentration of 2.25µg/ml in a 150µl medium volume. Absorbance at 450 nm was measured 4h later using Asys UVM 340 microplate reader (Biochrom). CellTiter-Blue was used according to the manufacturer’s protocol: 20µl of reagent was added per 100µl of medium and fluorescence was measured 4h later. Black opaque plates were used for fluorescence readings using a Victor X4 instrument (Perkin Elmer). Additional wells in tetraplicates with culture medium and without cells were used to measure background absorbance/fluorescence. Dasatinib sensitivity was calculated as the relative absorbance/fluorescence of treated vs untreated cells after subtraction of background fluorescence.

CLL cells from some patients (n=8) were assayed using both the XTT assay and the CellTiter-Blue method, which proved to be concordant in our settings (Supplementary Figure S1). The more sensitive CellTiter-Blue method was therefore used as an additional indicator of dasatinib sensitivity in inconclusive cases (Figure 1A).

**RNA library preparation and sequencing**

Simultaneously with dasatinib sensitivity assay, the cells were seeded for further RNA isolation into 6-well plates at 10 × 10^6^ cells / 8ml and exposed to dasatinib at a final concentration of 180nM or IMDM medium only. After 48h, the cells were rinsed with PBS and centrifuged. RNA was isolated with TriPure Isolation Reagent (Roche) and chloroform and dissolved in water. RNA quality and concentration were assessed using Qubit RNA Broad Range Assay (Thermo Fisher) and RNA 6000 Nano (Agilent) kits.

Libraries were prepared from 1μg of total RNA per sample, as detailed in the TruSeq RNA Library Prep Kit v2 (Illumina, San Diego, CA, USA). Samples were sequenced on the Illumina HiSeq2000 to generate 100bp paired-end reads, with a mean read depth of 63 million read pairs (range: 51-81 million).

**RNA sequencing data analysis**

Reads were aligned to the human reference genome hg19 using STAR’s two-pass alignment.^2^ with transcriptome annotation from GENCODE release 19.^3^ Differentially expressed genes (DEGs) with an absolute fold change ≥ 1.5 (log2 fold change ≥ 0.6) at a false discovery rate (FDR) ≤ 0.1 were determined using DESeq2.^4^ Principal component analyses were also performed using DESeq2; hierarchical clusterings and heatmaps were generated using the R package pheatmap.^5^ Gene set enrichment analysis was performed using GSEA and the hallmark gene set collection of the MSigDB^6^, genes were pre-ranked based on the log2 fold change estimates from DESeq2. To identify drug effects correlated with specific gene-expression changes, up- and down-regulated DEGs were separately entered at [http://amp.pharm.mssm.edu/L1000CDS2](http://amp.pharm.mssm.edu/L1000CDS2/#/index%23/index%23/index%23/index) to query the LINCS L1000 database.^7^ From the top 50 compounds returned, we filtered duplicates, retaining the top scoring entry, and excluded experimental compounds with numerical names. The unfiltered lists of compounds are provided in Figure S3. For variant calling, alignments were processed by Picard to remove duplicates (<http://broadinstitute.github.io/picard/>) and locally realigned by the GATK.^8^ Pileup files were created using SAMtools^9^ and passed to VarScan2 for variant calling.^10^ Variants were then annotated by ANNOVAR^11^ and filtered as follows: non-synonymous missense, nonsense, insertion and deletion mutations affecting exonic regions as well as all mutations affecting splice sites were extracted. Of these, we retained variants supported by at least 4 reads in total, at least one forward and reverse read and a variant allele frequency (VAF) > 10%. We removed those residing in regions of segmental duplication and those annotated in dbSNP^12^ but not in COSMIC^13^ as well as those annotated as benign in dbSNP and ClinVar.^14^ The remaining variants were further filtered to discard those with a frequency above 1% in healthy populations (esp6500, 1000G). Mutations were then reported for a given patient if they were supported by both the treated and untreated sample of each patient and if results were concordant with those obtained from DNA sequencing (see below), which for this cohort they were in all cases. The resulting mutations were each on average covered by 106 sequencing reads.

Copy-number alterations were detected by sequencing coverage analysis using CNVkit v0.9.5^15^ and again only reported if results were concordant with RNA sequencing results (see above). We note that the resulting CNVs were also concordant with available FISH data in 8/9 cases; in one responder –11q was reported by FISH in <20% of cells, likely below the sequencing-based limit of detection.

All of the scripts used for analyses are freely available for download and inspection at <https://github.com/tjblaette/ngs>. Normalized gene expression values and raw sequencing data can be accessed via Gene Expression Omnibus (GEO accession number GSE151159).

**DNA library preparation and sequencing**

Mutational background was determined through targeted sequencing of approximately 1.1Mb of exonic regions of hematologic malignancy-associated genes (Table S4). DNA was isolated before dasatinib treatment from 200µl of fresh peripheral blood using DNA Blood Mini Kit (Qiagen). Genomic DNA libraries were constructed using 500ng of DNA and KAPA HyperPlus library preparation kit, enriched in 26-plex pool using SeqCap EZ custom capture (Roche) and sequenced on Illumina NovaSeq 6000 platform (2 × 100nt reads), reaching a median 344.79× mean coverage (range 285.46 – 426.05×) and median 97.3% ge20 (range 97.2– 97.3%). All steps were carried out according to original protocols with minor modifications.

**DNA sequencing data analysis**

Variant discovery included following steps: quality control of raw fastq, adapter trimming and low quality reads removal using Trimmomatic^16^, read mapping to hg19 genome using BWA^17^, duplication removal, local realignment and quality recalibration using GATK and Picard and variant calling using HaplotypeCaller^18^ . Variants with VAF ≥ 5% were filtered using gnomAD^19^ and internal database in order to remove common genetic variation, using 1% and 5% allele frequency thresholds, respectively. Selected variants were then manually curated using ClinVar^20^ and Varsome ^21^ databases.

Copy-number calling was performed by sequencing coverage analysis using CNVkit v0.9.5 ^15^ . A group of 15 CLL samples without copy number variations (CNVs) was used to create reference coverage model. CNVkit was run with default settings except that bin size was limited to a maximum of 400bp.

**Supplementary References**

1. Takahashi N, Miura M, Scott SA, Niioka T, Sawada K. Pharmacokinetics of dasatinib for Philadelphia-positive acute lymphocytic leukemia with acquired T315I mutation. *Journal of Hematology & Oncology.* 2012;5(1)**:** 23.

2. Dobin A, Davis CA, Schlesinger F et al. STAR: ultrafast universal RNA-seq aligner. *Bioinformatics.* 2013;29(1)**:** 15-21.

3. Harrow J, Frankish A, Gonzalez JM et al. GENCODE: the reference human genome annotation for The ENCODE Project. *Genome Res.* 2012;22(9)**:** 1760-1774.

4. Love MI, Huber W, Anders S. Moderated estimation of fold change and dispersion for RNA-seq data with DESeq2. *Genome Biol.* 2014;15(12)**:** 550.

5. Kolde R. pheatmap: Pretty Heatmaps. R package version 1.0.8. 2015. In, 2015.

6. Subramanian A, Tamayo P, Mootha VK et al. Gene set enrichment analysis: a knowledge-based approach for interpreting genome-wide expression profiles. *Proc Natl Acad Sci U S A.* 2005;102(43)**:** 15545-15550.

7. Duan Q, Reid SP, Clark NR et al. L1000CDS(2): LINCS L1000 characteristic direction signatures search engine. *NPJ Syst Biol Appl.* 2016;2.

8. DePristo MA, Banks E, Poplin R et al. A framework for variation discovery and genotyping using next-generation DNA sequencing data. *Nat Genet.* 2011;43(5)**:** 491-498.

9. Li H, Handsaker B, Wysoker A et al. The Sequence Alignment/Map format and SAMtools. *Bioinformatics.* 2009;25(16)**:** 2078-2079.

10. Koboldt DC, Zhang Q, Larson DE et al. VarScan 2: somatic mutation and copy number alteration discovery in cancer by exome sequencing. *Genome Res.* 2012;22(3)**:** 568-576.

11. Wang K, Li M, Hakonarson H. ANNOVAR: functional annotation of genetic variants from high-throughput sequencing data. *Nucleic Acids Res.* 2010;38(16)**:** e164.

12. Sherry ST, Ward MH, Kholodov M et al. dbSNP: the NCBI database of genetic variation. *Nucleic Acids Res.* 2001;29(1)**:** 308-311.

13. Forbes SA, Beare D, Boutselakis H et al. COSMIC: somatic cancer genetics at high-resolution. *Nucleic Acids Res.* 2017;45(D1)**:** D777-D783.

14. Landrum MJ, Lee JM, Benson M et al. ClinVar: public archive of interpretations of clinically relevant variants. *Nucleic Acids Res.* 2016;44(D1)**:** D862-868.

15. Talevich E, Shain AH, Botton T, Bastian BC. CNVkit: Genome-Wide Copy Number Detection and Visualization from Targeted DNA Sequencing. *PLoS Comput Biol.* 2016;12(4)**:** e1004873.

16. Bolger AM, Lohse M, Usadel B. Trimmomatic: a flexible trimmer for Illumina sequence data. *Bioinformatics.* 2014;30(15)**:** 2114-2120.

17. Li H. Aligning sequence reads, clone sequences and assembly contigs with BWA-MEM. Available from: URL (Accessed n Date Accessed Year)|.

18. Van der Auwera GA, Carneiro MO, Hartl C et al. From FastQ data to high confidence variant calls: the Genome Analysis Toolkit best practices pipeline. *Curr Protoc Bioinformatics.* 2013;43(1110)**:** 11.10.11-11.10.33.

19. Lek M, Karczewski KJ, Minikel EV et al. Analysis of protein-coding genetic variation in 60,706 humans. *Nature.* 2016;536(7616)**:** 285-291.

20. Landrum MJ, Lee JM, Benson M et al. ClinVar: improving access to variant interpretations and supporting evidence. *Nucleic Acids Res.* 2018;46(D1)**:** D1062-d1067.

21. Kopanos C, Tsiolkas V, Kouris A et al. VarSome: the human genomic variant search engine. *Bioinformatics.* 2019;35(11)**:** 1978-1980.

**Supplementary Figures**

**Figure S1:** Comparison of XTT and CellTiterBlue viability assays results for patients treated with dasatinib 180nM and analyzed with both methods. Neighboring data points represent viability measurement with both methods for individual patients. Error bars represent a fraction of standard deviation in treated cells vs mean viability measurement in corresponding controls.


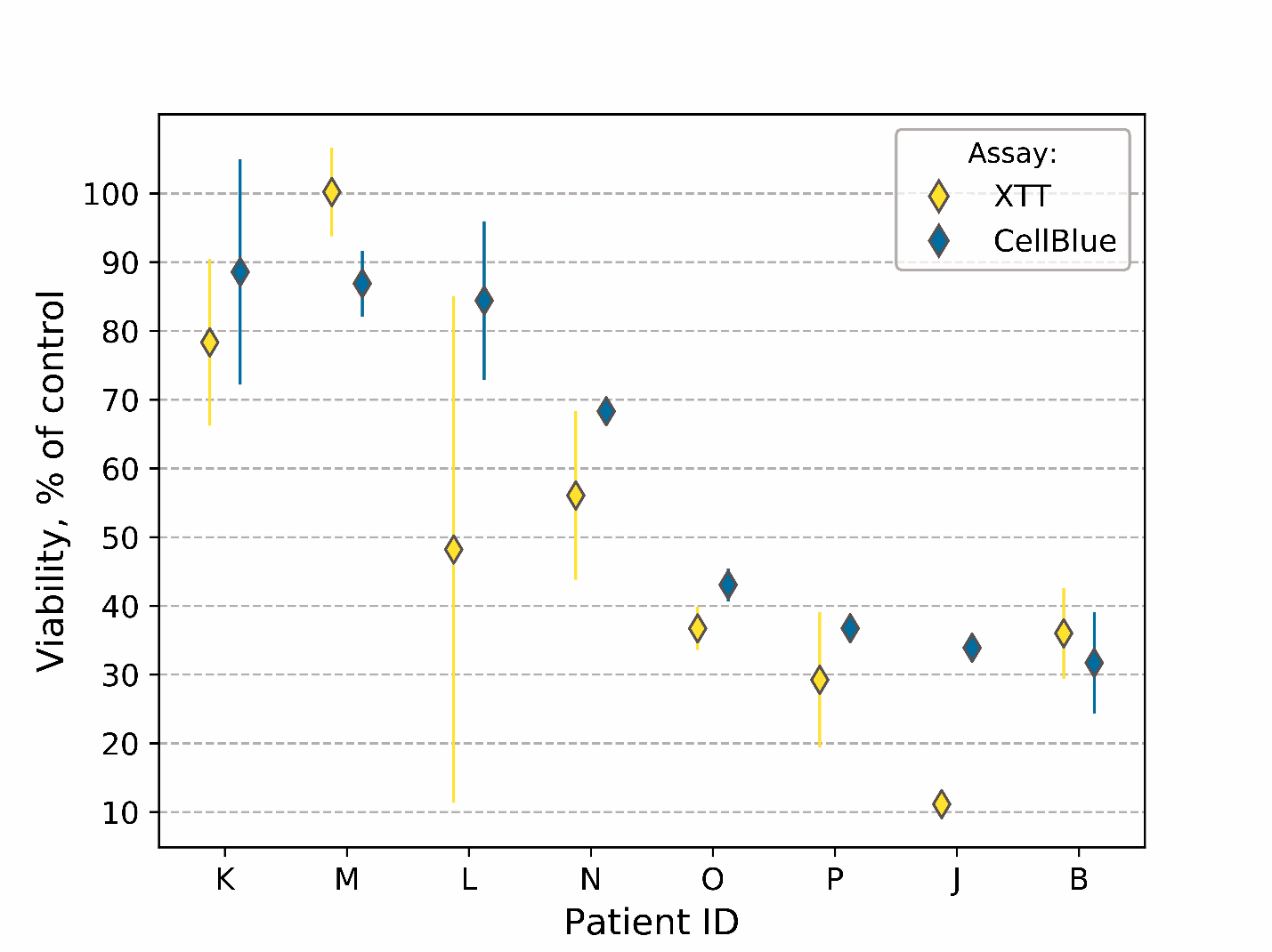


**Figure S2:** Drug effects correlated with specific gene-expression changes between treated and untreated samples **(A)** and responders and non-responders **(B)**. Differentially expressed genes (DEGs) determined in our cohort were matched with those differentially expressed in cell lines upon treatment with various drugs, using the LINCS L1000 database. Drugs whose effect correlated with those of dasatinib treatment in our cohort returned several kinase inhibitors, including dasatinib itself, validating the gene expression signatures that we observe in our data **(A)**. Drugs anti-correlated with dasatinib resistance in our cohort are speculated to sensitize non-responders to dasatinib **(B)**. Compounds shown here are filtered; refer to Supplementary Figure S3 for the original top 50 hits returned by the LINCS L1000 database.


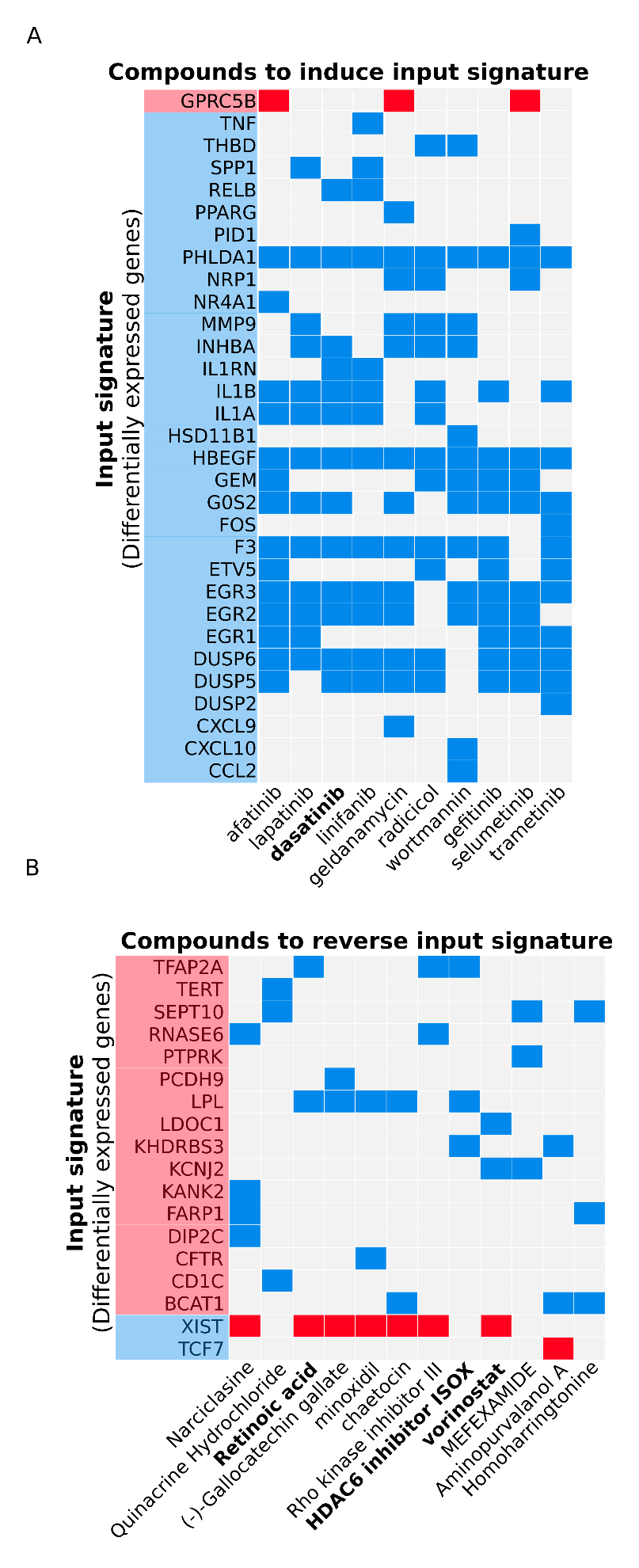


**Figure S3:** The original, unfiltered top 50 hits returned by the LINCS L1000 database, which were further filtered to generate corresponding figures S2A and S2B.


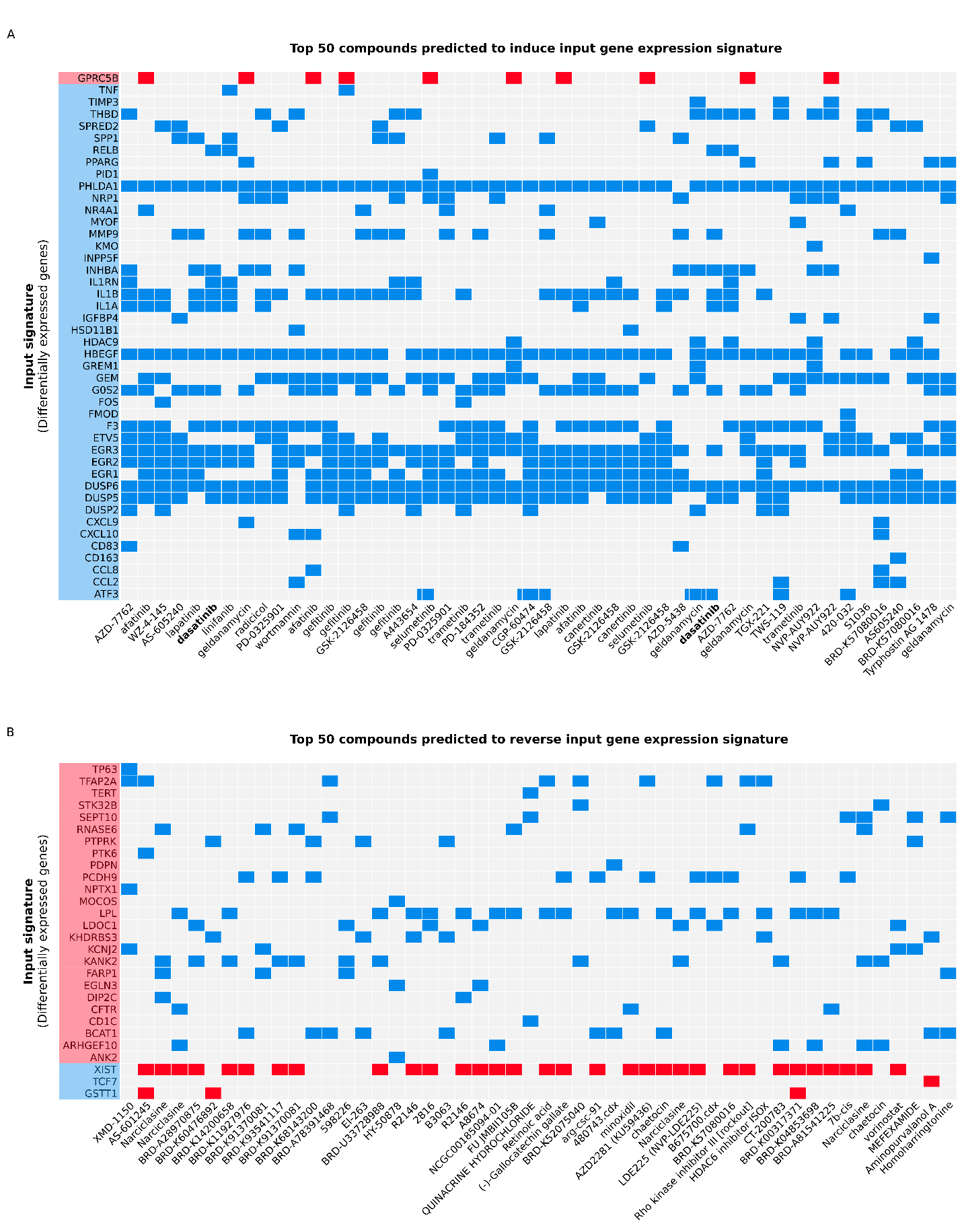


**Supplementary Tables**

**Table S1:** Baseline clinical characteristics of CLL patients

|  | Responders  N=7 (100%) | Non-responders  N=9 (100%) | Total N=16 (100%) |
| --- | --- | --- | --- |
| Sex  Male  Female | 5 (71.0%)  2 (29.0%) | 3 (33.3%)  6 (66.7%) | 8 (50.0%)  8 (50.0%) |
| Age  Mean  Median  Range | 68.2  71  47-84 | 66.7  66  57-78 | 67.3  67  47-84 |
| Rai stage  0  1  2  3  4 | 1 (14.5%)  2 (28.5%)  2 (28.5%)  -  2 (28.5%) | 6 (66.7%)  2 (22.2%)  -  1 (11.1%)  - | 7 (43.8%)  4 (25.0%)  2 (12.5%)  1 (6.3%)  2 (12.5%) |
| WBC (x10^9^/L)  Mean  Median  Range | 99.89  59.95  16.35-381.53 | 28.75  19.28  13.06-58.37 | 59.87  35.86  13.06-381.53 |
| CD5 (%)  Mean  Median  Range  Not available | 69.26  76.02  21.72-93.20  1 | 73.43  75.50  33.39-87.72  - | 71.76  75.5  21.79-93.20  1 (6.3%) |
| PLT (x10^9^/L)  Mean  Median  Range | 158.86  166  62-253 | 164.56  137  116-252 | 162.06  142.5  62-253 |
| IGHV  Mutated  Unmutated | 1 (14.5%)  6 (85.5%) | 8 (88.9%)  1 (11.1%) | 9 (56.3%)  7 (43.8%) |
| ZAP-70+   (cutoff 20%) | 1 (14.5%) | 1 (11.1%) | 2 (12.5%) |
| FISH  Normal  del11q/ATM  del17p/TP53  Not available | 3  1  -  3 | 5  -  -  4 | 8 (50.0%)  1 (6.3%)  0  7 (43.8%) |

**Table S2:** Differentially expressed genes (DEGs) identified between all responder vs all non-responders samples (left column), treated responder vs treated non-responder samples (middle column) and untreated responder vs untreated non-responder samples (right column). Gene symbols are alphabetically sorted and arranged to show both overlapping and distinct DEGs of each comparison.

| All responders vs non-responders  (n=154 DEGs) | Treated responders vs non-responders  (n=20 DEGs) | Untreated responders vs non-responders  (n=31 DEGs) |
| --- | --- | --- |
| AC005237.4  AC005307.3  AC005498.4  AC005537.2  AC007952.5  AC008132.13  AC023490.1  AC061992.2  AC096579.7  ADAM29  ADAMTS7  ANGPT2  ANK2  ANKRD13B  AP000350.5  AP000351.10  AP001257.1  ARHGAP23  ARHGAP39  ARHGEF10  ARSD  BCAT1  BTNL9  CD1C  CFTR  CLEC4C  CTC-508F8.1  CTD-2231H16.1  CTTNBP2  DBNDD1  DIP2C  DLX4  DNAH14  DNAH3  DPF3  DRP2  DTX1  EFCAB4A  EGLN3  FAM174B  FARP1  FER1L4  FRMD5  GGT3P  GNAO1  GPER1  GPR173  GSTT1  IGHV1-2  IGHV1-3  IGHV1-69  IGHV4-55  IGKV1-12  ILDR1  ITPKA  KANK2  KB-1592A4.15  KB-226F1.1  KBTBD12  KCNJ2  KCNJ2-AS1  KHDRBS3  KLK4  KLKP1  LDOC1  LINC00930  LPL  LTK  MAP2K6  MAPK12  MAPK4  MGAT3  MID1IP1-AS1  MIR4458HG  MOCOS  MRO  MS4A4E  MYO3A  MYOM2  NAT8L  NETO1  NPTX1  NUGGC  OR1H1P  OSTN  PAX9  PCDH9  PDPN  PIEZO2  PLEKHG4B  POM121L10P  POM121L9P  POU3F3  PTK6  PTPRK  RBM20  RBMS3  RGS11  RHBDF1  RIMBP2  RLN2  RN7SKP275  RNASE6  RP11-1070N10.5  RP11-1070N10.7  RP11-134N1.2  RP11-162O12.2  RP11-259G18.3  RP11-281P23.1  RP11-386M24.3  RP11-386M24.6  RP11-388M20.9  RP11-394O9.1  RP11-417P24.1  RP11-430H10.1  RP11-441F2.2  RP11-480D4.2  RP11-480D4.6  RP11-707O23.5  RP11-713C5.1  RP11-798K23.5  RP4-801G22.2  RP4-801G22.3  RP5-1063M23.2  RPL17P46  RPS6KA6  RTKN  SAMD12  SAMD7  SDC3  SEPT10  SESN3  SH2D4A  SHISA3  SLAMF1  SLC16A9  SNED1  STK32B  TCF7  TERT  TFAP2A  TFAP2A-AS1  TLE6  TP63  TRIM72  UTS2B  WNT9B  XIST  Y  ZNF334  ZNF469  ZNF471  ZNF667  ZNF667-AS1 | ADAM29  ARHGEF10  IGHV1-69  ITPKA  LINC00930  LPL  MAPK4  MIR4458HG  MS4A4E  NETO1  PLEKHG4B  RBMS3  RP11-134N1.2  RP11-386M24.6  RP11-480D4.2  RP11-480D4.6  SAMD12  STK32B  ZNF667  ZNF667-AS1 | AC008132.13  AC096579.7  ADAM29  ANK2  ARHGEF10  BTNL9  CD1C  CTTNBP2  DNAH14  FRMD5  IGHV1-69  ILDR1  ITPKA  KCNJ2  KCNJ2-AS1  LPL  MAPK4  MIR4458HG  MS4A4E  NETO1  PLEKHG4B  PTK6  RBMS3  RP11-134N1.2  RP11-480D4.2  RP11-480D4.6  RP5-1063M23.2  SAMD7  TP63  ZNF667  ZNF667-AS1 |

**Table S3:** Gene mutations in genes TP53, ATM, SF3B1, NOTCH1, BIRC3 and MYD88 called from RNA and DNA sequencing data as well as copy-number variations (CNVs) called from RNA and sequencing data or FISH. Both gene mutations and CNVs were only reported when they were concordant between respective assays and supported by data from both the treated and untreated sample of a given patient to reduce false positives.

|  | Responders  N=7 (100%) | Non-responders  N=9 (100%) | Total N=16 (100%) |
| --- | --- | --- | --- |
| Gene mutation  ATM  BIRC3  NOTCH1  SF3B1  MYD88  TP53 | 1 (14%)  2 (29%)  1 (14%)  2 (29%)  0  0 | 1 (11%)  0  1 (11%)  0  0  0 | 2 (12.5%)  2 (12.5%)  2 (12.5%)  2 (12.5%)  0  0 |
| Copy-number variation  +2p  del(4q34-35)  del11q/ATM  +12  del(13q11-q21.33)  del17p/TP53 | 1 (14%) 0  2 (29%)  2 (29%)  0  0 | 0 1 (11%)  0  0  1 (11%)  0 | 1 (6.3%)  1 (6.3%)  2 (12.5%)  2 (12.5%)  1 (6.3%)  0 |

**Table S4:** List of genes sequenced as part of the DNA sequencing panel (gene names according to RefSeq Genes).


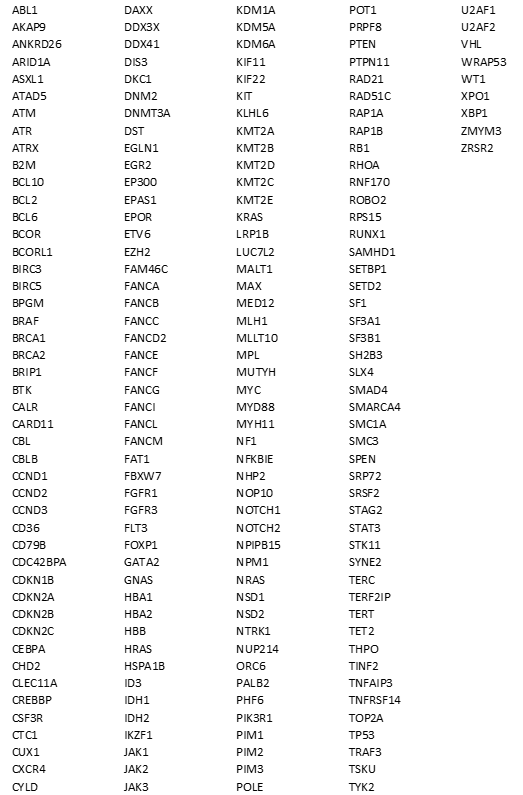

Supplement: Supplementary file 1 [file hs9-5-e514-s001.docx]
